# Dermatomyosite amyopathique révélant un carcinome thymique

**DOI:** 10.11604/pamj.2015.21.292.6887

**Published:** 2015-08-21

**Authors:** Madiha Mahfoudhi, Khaled Khamassi

**Affiliations:** 1 Service de Médecine Interne A, Hôpital Charles Nicolle, Tunis, Tunisie; 2 Service ORL, Hôpital Charles Nicolle, Tunis, Tunisie

**Keywords:** Dermatomyosite amyopathique, érythème du visage, dyspnée récente, Amyopathic dermatomyositis, facial erythema, dyspnoea

## Image en medicine

La dermatomyosite amyopathique est une entité rare caractérisée par des lésions cutanées suggestives de dermatomyosite sans atteinte musculaire. Dans les formes paranéoplasiques qui sont rares, le pronostic dépend du type et de la réponse thérapeutique de la tumeur associée. L'association à un carcinome thymique est exceptionnelle. Un patient âgé de 50 ans a été hospitalisé pour exploration d'un érythème du visage et une dyspnée récente. L'examen physique a révélé un érythème héliotrope du visage, des papules de Gottron en regard des articulations interphalangiennes et un érythème douloureux péri-unguéal. Le reste de l'examen était sans particularités. L'examen biologique a montré un syndrome inflammatoire. Le dosage des enzymes musculaires était normal. L'examen immunologique était négatif. L'électromyogramme était sans anomalies. Une biopsie musculaire a montré des fibres musculaires striées normales. Le diagnostic de dermatomyosite amyopathique a été retenu. Dans le cadre de la recherche d'une tumeur associée (digestive, pulmonaire ou gynécologique), les échographies cervicale, abdominale et pelvienne étaient normales. La fibroscopie digestive haute et la coloscopie n'ont révélé aucune anomalie. La TDM thoraco-abdomino- pelvienne a montré une image médiastinale antérieure hétérogène mesurant 92 mm x 40 mm et se réhaussant de façon intense après injection du produit de contraste . Le patient a été opéré par sternotomie permettant une excision complète de la masse. L'examen anatomopathologique a conclu à un carcinome épidermoïde du thymus. Une chimiothérapie et une radiothérapie centrée sur le sternum ont été instaurées. L'évolution était favorable sans notion de récidive avec un recul de trois ans.

**Figure 1:**
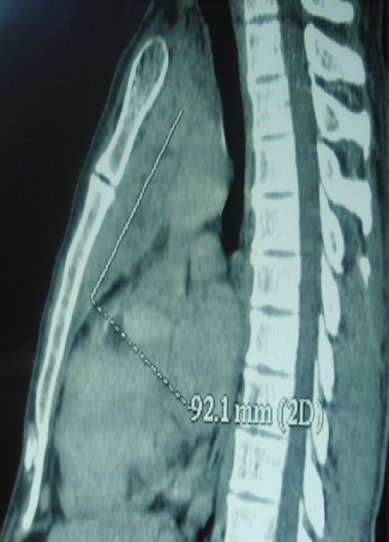
TDM thoracique (reconstruction sagittale), masse médiastinale antérieure mesurant 92 mm x 40 mm

